# Involvement of Hdac3-mediated inhibition of microRNA cluster 17-92 in bronchopulmonary dysplasia development

**DOI:** 10.1186/s10020-020-00237-4

**Published:** 2020-11-03

**Authors:** Di Wang, Hui Hong, Xiao-Xia Li, Jing Li, Zhi-Qun Zhang

**Affiliations:** 1 Department of Pediatrics, Affiliated Hangzhou First People’s Hospital, Zhejiang University School of Medicine, Hangzhou, 310006 People’s Republic of China; 2 Department of Neonatology, Affiliated Hangzhou First People’s Hospital, Zhejiang University School of Medicine, No. 261, Huansha Road, Hangzhou, 310006 Zhejiang People’s Republic of China

**Keywords:** Histone deacetylase 3, microRNA-17-92 cluster, Placental growth factor, Bronchopulmonary dysplasia, Pulmonary angiogenesis, Alveolarization

## Abstract

**Background:**

The incidence of bronchopulmonary dysplasia (BPD), a chronic lung disease of newborns, has been paradoxically rising despite medical advances. Histone deacetylase 3 (Hdac3) has been reported to be a crucial regulator in alveologenesis. Hence, this study aims to investigate the mechanism of Hdac3 in the abnormal pulmonary angiogenesis and alveolarization of BPD.

**Methods:**

A hyperoxia-induced BPD model of was developed in newborn mice, and primary lung fibroblasts were isolated from adult mice. Hdac3 was knocked out in vivo and knocked down in vitro, while microRNA (miR)-17 was downregulated in vivo and in vitro to clarify their roles in abnormal pulmonary angiogenesis and alveolarization. Mechanistic investigations were performed on the interplay of Hdac3, miR-17-92 cluster, enhancer of zeste homolog 1 (EZH1), p65 and placental growth factor (Pgf).

**Results:**

Hdac3 was involved in abnormal alveolarization and angiogenesis in BPD mice. Further, the expression of the miR-17-92 cluster in BPD mice was downregulated by Hdac3. miR-17 was found to target EZH1, and Hdac3 rescued the inhibited EZH1 expression by miR-17 in lung fibroblasts. Additionally, EZH1 augmented Pgf expression by recruiting p65 thus enhancing the progression of BPD. Hdac3 augmented the recruitment of p65 in the Pgf promoter region through the miR-17/EZH1 axis, thus enhancing the transcription and expression of Pgf, which elicited abnormal angiogenesis and alveolarization of BPD mice.

**Conclusions:**

Altogether, the present study revealed that Hdac3 activated the EZH1-p65-Pgf axis through inhibiting miR-17 in the miR-17-92 cluster, leading to accelerated abnormal pulmonary angiogenesis and alveolarization of BPD mice.

## Background

Bronchopulmonary dysplasia (BPD) is a common chronic lung disease characterized by arrested alveolar development or loss of alveoli which commonly occurs in premature infants. BPD is associated with increased risks of morbidity and mortality, as well as adverse neurodevelopmental outcomes among prematurely born infants (Gallini et al. 2014; Thebaud and Abman 2007). Risk factors responsible for BPD include pre- and postnatal infections, hyperoxia, as well as mechanical ventilation, which not only influence the interacted function of pro- and anti-inflammatory proteins, but also the extra alterations of signaling pathways which is closely related to the imbalance of growth factor (Niedermaier and Hilgendorff 2015). Although extensive approaches have improved the survival of preterm infants in neonatal care, the effectiveness of the currently available therapeutic strategies in reducing the incidence and severity of BPD remains very limited (Davidson and Berkelhamer 2017). Therefore, it is important to determine the specific molecular mechanism of BPD in order to identify potential novel biomarkers relevant to BPD.

Histone deacetylases (HDACs) are chromatin-modifying enzymes that have emerged as the important targets for treating various diseases due to their powerful regulatory role in physiological and pathological settings (Cantley et al. 2017). Hdac3, a member of the HDAC superfamily, has been shown to play a pivotal role in cellular proliferative and apoptotic potential as well as transcriptional repression, and may cause a decline in angiogenesis (Park et al. 2014). However, the regulatory function of Hdac3 in BPD remains unknown. Evidence has shown that the knockdown of Hdac3 could upregulate the expression of microRNA (miR)-15a/16-1, thereby suppressing lung cancer cell growth and colony formation (Chen et al. 2013). miRNAs are a class of small noncoding RNAs that regulate gene expression at the post-transcriptional level, leading to mRNA degradation or inhibition of protein translation (Singh et al. 2013). The miR-17-92 cluster (miR-17, miR-18a, miR-19a, miR-19b, miR-20a, and miR-92) has been reported to be poorly expressed in lung tissues of BPD infants, while the downregulation of miR-17-92 cluster was closely related to the development of BPD (Rogers et al. 2015).

Interestingly, miR-17 has been identified to target enhancer of zeste homolog 1 (EZH1) and downregulates its expression, sensitizes non-small cell lung cancer cells to erlotinib (Zhang et al., 2017). Additionally, the binding of EZH1 to the p65 transcription factor could augment the transcription of downstream target genes (Su et al. 2016). Evidence exists suggesting that p65 could directly bind to the promoter region of placental growth factor (Pgf)/Plgf and transcriptionally activates the expression of Pgf/Plgf (Cramer et al. 2005). Pgf is a member of the vascular endothelial growth factor (VEGF) family of angiogenesis regulators and the molecular structure is glycoprotein homodimer; higher plasma VEGF/Pgf levels have been found in preterm neonates with BPD and in those who died before 28 days of life, indicating an important role of this substance in pulmonary vascular development (Procianoy et al., 2016). Moreover, a previous animal model study has confirmed that Pgf overexpression impairs lung development in newborn rats, while its inhibition using an anti-PGF antibody ameliorates the lung impairment (Zhang et al., 2020). Therefore, it was hypothesized that EZH1 may mediate p65-activated transcription of Pgf in BPD mice. In the present work, we planned to explore the potential effects of Hdac3 regulating miR-17 in the miR-17-92 cluster on the abnormal angiogenesis and alveolarization of BPD via EZH1-p65-Pgf axis, which may serve as a potential target to treat BPD.

## Methods

### Ethics statement

All animal experiments were conducted in strict accordance with the Guide for the Care and Use of Laboratory animals published by the US National Institutes of Health. The protocol of animal experiments was approved by the Institutional Animal Care and Use Committee of Affiliated Hangzhou First People’s Hospital, Zhejiang University School of Medicine. All efforts were made to minimize the number and suffering of the included animals.

### Hyperoxia-induced BPD mouse model establishment

Fifty C57BL/6 J newborn mice (within 2 h of birth) with an average weight of 1.36 g ± 0.09 g were randomly divided into one litter (equal numbers of mice per litter). The mice for BPD modeling were exposed to 85% oxygen for postnatal day 1 (P1)—P14, while the control mice with normal lung development were exposed to 21% oxygen. Male and female animals were used because no gender bias was found in the study on perturbations to lung development of C57BL/6 J mice in response to hyperoxia. The normal hypoxia and hyperoxia was alternated every 24 h in order to limit oxygen toxicity. The mice were then randomly assigned into the control group (normal air-treated C57BL/6 J newborn mice), the BPD group (hyperoxia-induced BPD mice), the BPD + Hdac3 knockout (Hdac3^−/−^) group (BPD mice treated with Hdac3 knockout) (Wang et al. 2016), the hyperoxia + Hdac3^−/−^ + antagomir negative control (NC) group (hyperoxia-induced BPD mice with Hdac3 knockout and injected with antagomir NC), and the hyperoxia + Hdac3^−/−^ + miR-17-antagomir group (hyperoxia-induced BPD mice with Hdac3 knockout and injected with miR-17-antagomir), with ten mice in each group. From the 5th day following grouping, mice were intraperitoneally injected with miR-17-antagomir and the corresponding antagomir NC (both were purchased from Shanghai GenePharma Co., Ltd., Shanghai, China) at a dose of 80 mg/kg body weight, after which the weight was measured every three days. Mice in P14 (the stage of massive alveolarization) were euthanized by intraperitoneal injection of excessive pentobarbital (500 mg/kg), and the lungs of mice were then extracted for further analyses (Zhu et al., 2020).

### Cell culture and grouping

Primary lung fibroblasts were isolated from C57BL/6 J mice. Briefly, the lungs were injected with approximately 500 μL of preheated collagenase type I (2 mg/mL) at 37 °C and then excised from adult female C57BL/6 J mice after being euthanized by isoflurane inhalation. The lung tissues were then placed in preheated collagenase type I and treated at 70 rpm for 1 h at 37 °C using an orbital rotator (Unimax 1010). The lungs were then minced and the tissue suspension was dispersed by using a 20G syringe needle. The cell suspension was then centrifuged to discard supernatant. The cell pellet was then resuspended in preheated (37 °C) high-glucose Dulbecco's modified Eagle's medium (DMEM) containing 10% (v/v) fetal bovine serum (FBS), 100 U/mL penicillin (Thermo Fisher Scientific, Waltham, MA) and 100 μg/mL streptomycin (Thermo Fisher Scientific, Waltham, MA), and then passaged in low-glucose DMEM containing 10% (v/v) FBS, 100 U/mL penicillin, and 100 μg/mL streptomycin. The cells used in this study were primarily isolated and cultured lung fibroblasts (Ruiz-Camp et al. 2019).

The lung fibroblasts were cultured in a 6-well plate at a cell concentration of 2 × 10^5^ cells per well. When the cells reached 80% confluence, transfection was performed based on the manufacturer’s protocols of the Lipofectamine 2000 reagents (11668-019, Invitrogen, New York, California). The fibroblasts were transfected with following plasmids: short hairpin (sh) RNA-NC, sh-Hdac3, inhibitor-NC, miR-17-inhibitor, sh-Hdac3 + inhibitor-NC, sh-Hdac3 + miR-17-inhibitor, overexpression (oe)-NC, oe-EZH1, sh-NC, sh-p65 and oe-EZH1 + sh-p65 (Guangzhou RiboBio Co., Ltd., Guangzhou, Guangdong, China). After 12 h of transfection, the cells were cultured for 48 h at 37 °C under 5% CO_2_ for subsequent RNA extraction and other relevant experiments.

### Hematoxylin–eosin (HE) staining

The neutral formaldehyde-fixed, paraffin-embedded sections of mouse left lung tissues were dewaxed, and then rehydrated. stained with hematoxylin. Subsequently, the sections were stained with eosin, hydrated, permeabilized, and air-dried. The sections were then mounted with neutral resin and the morphological changes of lung tissues were observed under an optical microscope.

### Immunohistochemistry

Left lung tissues were fixed in 4% paraformaldehyde, embedded in paraffin, serially sectioned at a thickness of 4 μm and routinely dewaxed. The streptavidin-peroxidase method was routinely conducted. Next, antigen retrieval was performed by microwave heating. The sections were blocked by the addition of normal goat serum blocking solution. The staining was conducted with the HistostainTM SP-9000 immunohistochemical staining kit (Zymed Laboratories Inc., South San Francisco, CA). The sections were then probed at 4 °C overnight with the following primary antibodies purchased from Abcam Inc.: rabbit anti-EZH1 (ab64850, 1: 100), rabbit anti-NF-κB p65 (ab16502, 1: 100), and rabbit anti-Pgf (ab230516, 1: 100), followed by probing with horseradish peroxidase-labeled mouse anti-rabbit secondary antibody (ab6728, 1: 1000, Abcam, Inc., Cambridge, UK) at 37 °C for 30 min. After DAB development, the sections were subjected to hematoxylin counterstaining and sealed using gum. Then, five representative high-power fields were selected for cell observation and counting. The cytoplasm stained brown or yellow were indicative of positive cells.

### Immunofluorescence staining of microvessel density (MVD)

The lung tissue sections were deparaffinized, rehydrated, and stained with rabbit polyclonal antibody to Von Willebrand Factor (vWF, ab11713, 1: 100, Abcam, Inc). The number of blood vessels (20–50 μm diameter) of each high power field was counted in five randomly selected non-overlapping parenchymal regions of the lung tissue sections of the animals (*n* = 6). The detailed procedures were carried out as previously described (Reiter et al. 2017).

### RNA extraction and gene expression extraction

Lung fibroblasts in each group were collected and lysed using the TRIzol reagent (Invitrogen Inc., Carlsbad, CA). Then, the total RNA was extracted from cells and tissue samples. The quality and concentration of the extracted RNA were measured using an ultraviolet–visible spectrophotometer (ND-1000, Nanodrop, Wilmington, DE). An amount of 400 ng of the extracted RNA was subjected to reverse transcription using a PrimeScript RT Reagent Kit (Takara, Shiga, Japan). With complementary DNA (cDNA) as a template, a fluorescent quantitative PCR was carried out in accordance with the protocols provided of a SYBR® Premix Ex Taq™ II (Tli RNaseH Plus) kit (Takara). The primers were synthesized by RiboBio Company and are shown in Table 1. Glyceraldehyde-3-phosphate dehydrogenase (GAPDH) or U6 was used as a loading control gene. The fold changes were calculated using the relative quantification (the 2^−ΔΔCt^ method): ΔΔCT = Ct _(target gene)_—Ct _(loading gene)_ whereas ΔΔCt compares the experimental groups relative to the control group and would be ΔΔCt = ΔCt (experimental group)—ΔCt (control group).

**Table 1:** Primer sequences for RT-qPCR

Target	Primer sequence (5′-3′)
miR-17-92	F: TGAGGGCACTTGTAGCATTATG
	R: CCTTAGAACAAAAAGCACGCAG
miR-17-5p	F: GCGAATTCCAAATTTAGCAGGAATAAAG
	R: CGCTCGAGGACTGGACGCAGCCAGTG
Pgf	F: TTCCCCTTGGTTTTCCTCCTT R: AGATCTTGAAGATTCCCCCCA
GAPDH	F: AAATGGTGAAGGTCGGTGTGAACG
	R: ATCTCCACTTTGCCACTGC
U6	F: GAACCTCACCTTGGGACTGA
	R: TGCTACAAGTGCCCTCACTG

*F* forward, *R* reverse

### Western blot analysis

Lung fibroblasts underwent lysing were centrifuged to harvest supernatant, with protein concentration assessed using the bicinchoninic acid protein assay kit (Shanghai Beyotime Biotechnology Co. Ltd., Shanghai, China). Subsequently, 20 μg of protein was separated by 10% sodium dodecyl sulfate–polyacrylamide gel electrophoresis (SDS-PAGE) (Millipore, Billerica, MA) and transferred onto a polyvinylidene fluoride membrane (Millipore, Billerica, MA). The membrane was then blocked with 5% skimmed milk powder for 1 h and probed with the following Tris-buffered saline Tween-20 (TBST) diluted primary antibodies purchased from Abcam Inc., Cambridge, UK: rabbit polyclonal antibodies to Hdac3 (ab7030, 1: 1000), EZH1 (ab189833, 1: 250), p65 (ab16502, 1: 500) and Pgf (ab196666, 1: 1000) at 4 °C overnight, followed by 3 washes with TBST and probing with HRP-labeled secondary antibody goat anti-mouse or goat anti-rabbit (HS101, 1: 1000; TransGen Biotech Co., Ltd., Beijing, China) at room temperature for 1 h. Following 6 rinses with TBST, the immunocomplexes on the membrane were visualized using an enhanced chemiluminescence kit (Shanghai Baoman Biotechnology Co., Ltd., Shanghai, China). With GAPDH (ab37168, 1: 1000, Abcam, Inc) as a loading control, the band intensities were quantified using Image J analysis software.

### Chromatin immunoprecipitation (ChIP) assay

The enrichment of Hdac3 in the promoter region of the miR-17-92 cluster and that of p65 in the Pgf promoter region were evaluated by ChIP assay using the EZ-Magna ChIP TMA kit (Millipore). The lung fibroblasts were cross-linked with 1% formaldehyde. The cross-linked chromatin was sonicated to obtain 200–1000 bp chromatin fragments. DNA fragment underwent incubation with ChIP Dilution Buffer, pseudoisocyanine (PIC) and ProteinA agarose/salmon sperm DNA. After centrifugation, the supernatant was collected as the Input. Next, the supernatant in experimental group was subjected to incubation with rabbit anti-Hdac3 (ab7030, 1: 500, Abcam, Inc) and rabbit anti-p65 (ab19870, 1: 100, Abcam, Inc). The supernatant in the NC group underwent incubation with rabbit anti-immunoglobulin G (IgG) (ab172730, Abcam, Inc). The immunoprecipitated DNA was eluted with ChIP Wash Buffer and decrosslinked with NaCl, after which the DNA was recovered and the promoter sequences of miR-17-92 and Pgf in the complex were quantified by RT-qPCR.

### Dual-luciferase reporter gene assay

The constructed 3′untranslated region (3′UTR) luciferase vector of EZH1 was cloned into the pMIR reporter plasmid by PCR amplification of the potential binding site. For dual-luciferase reporter gene assay, based on Lipofectamine 2000 (Invitrogen Inc., Carlsbad, CA), lung fibroblasts containing the EZH1 3′UTR and miR-17-5p mimics and inhibitors were transiently transfected into the pMIR reporter vector. After 48 h of transfection, the luciferase activity was measured using a dual-luciferase assay system (Promega, Madison, WI). Renilla luciferase activity was used to normalize transfection efficiency. The promoter activity of HDAC for miR-17-92 cluster was described previously (Wang et al. 2016). The effect of p65 on the promoter activity of Pgf was described previously (Chaballe et al. 2011).

### Statistical analysis

Statistical analyses were conducted using SPSS 21.0 software (IBM Corp. Armonk, NY). Measurement data were expressed as mean ± standard deviation. Confirming to normal distribution and homogeneity of variance, the data between two groups with the unpaired design were compared using unpaired *t*-test. Data among multiple groups were analyzed by one-way analysis of variance (ANOVA), followed by a Tukey’s post-hoc test. The difference in *p*-value of *p* < 0.05 was considered as statistically significant.

## Results

### Abundant Hdac3 expression accelerates the progression of BPD

**Fig. 1:**
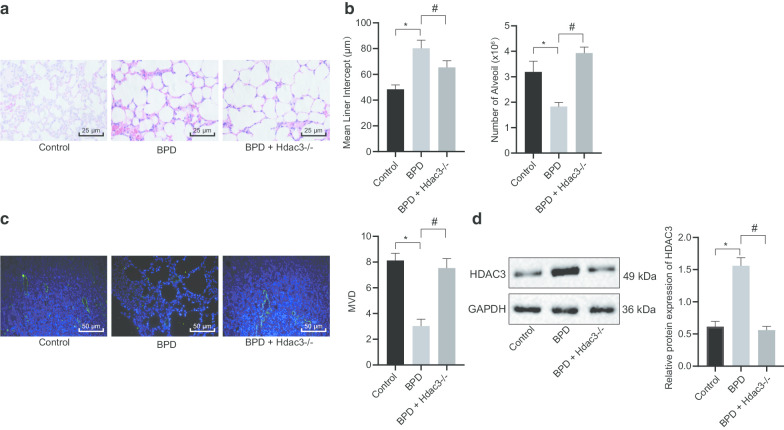
High Hdac3 expression facilitates abnormal alveolarization and angiogenesis in BPD mice. **a** Cell morphology of lung tissues of mice in each group assayed by HE staining (× 400). **b** The degree of abnormal alveolarization and MLI of alveoli in BPD mice. **c** MVD in lung tissues of mice in each group assessed by immunofluorescence assay (× 200). **d** Hdac3 protein expression in lung tissues of mice in each group assayed by Western blot analysis. *p < 0.05 compared to control mice. #*p* < 0.05 compared to untreated BPD mice. The above data were expressed as mean ± standard deviation. Data among multiple groups were analyzed by one-way ANOVA, followed by Tukey’s post-hoc test. *n* = 10 mice for each group

Previous literature has reported that Hdac3 participated in the remodeling and expansion of distant alveolar vesicles into primitive pulmonary alveolus by downregulating the miR-17-92 cluster (Wang et al. 2016), which commonly constitutes to the occurrence of BPD in premature infants (Ruiz-Camp et al. 2019). Based on the aforementioned evidence, it was suggested that Hdac3 may be involved in the progression of BPD. Therefore, the molecular mechanism of Hdac3 involved in BPD was explored by establishing a hyperoxia-induced mouse model of BPD. HE staining analysis suggested that the cell morphology was abnormal in the lung tissues of BPD mice relative to the control mice (Fig. 1a). The degree of abnormal alveolarization in BPD mice was subsequently examined and the results showed that the mean linear intercept (MLI) of alveoli in BPD mice was higher than that of the control mice, while the number of alveoli was less than that of the control mice (Fig. 1b). Furthermore, immunofluorescence assay suggested that the MVD of BPD mice was lower than that of the control mice (Fig. 1c). The Western blot analysis demonstrated that the expression of Hdac3 in lung tissues of BPD mice was markedly higher than that of control mice (Fig. 1d). Next, the Hdac3 gene was knocked out in BPD mice, and it was verified that the abnormal cell morphology was attenuated in BPD + Hdac3^−/−^ mice (Fig. 1a), the degree of abnormal alveolarization was notably lower (Fig. 1b), as well as the MVD was higher when compared to untreated BPD mice (Fig. 1c). The expression of Hdac3 in lung tissues of BPD + Hdac3^−/−^ mice was also lower than that in the BPD mice as assayed by Western blot analysis (Fig. 1d). These results demonstrated that Hdac3 may accelerate the abnormal alveolarization and angiogenesis in BPD.

### Hdac3 inhibits the expression of miR-17–92 cluster in BPD

**Fig. 2:**
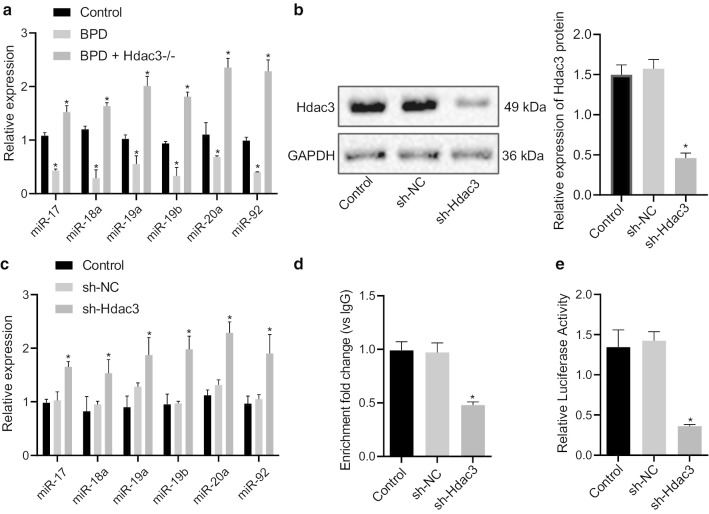
Hdac3 diminishes the expression of the miR-17-92 cluster in BPD mice. **a** The expression of miR-17-92 cluster in lung tissues of different groups of mice assayed by RT-qPCR normalized to U6. **b** Western blot analysis of Hdac3 protein expression in cells after silencing of Hdac3 expression. **c** The expression of miR-17-92 cluster in cells following Hdac3 silencing assayed by RT-qPCR normalized to U6. **d** The enrichment of Hdac3 in the promoter region of miR-17-92 cluster in cells following different transfection. **e** Luciferase activity of miR-17-92 cluster promoter assayed by dual-luciferase reporter gene assay. *p < 0.05 compared to control mice. The above data were expressed as mean ± standard deviation. Data among multiple groups were analyzed by one-way ANOVA, followed by Tukey’s post-hoc test. *n* = 10 mice for each group

Next, we moved to identify the expression of miR-17-92 cluster (miR-17, miR-18a, miR-19a, miR-19b, miR-20a, and miR-92) in lung tissues of mice in response to BPD modeling and Hdac3^−/−^. It was found that the expression of these miRNAs in lung tissues of BPD mice was lower than that in lung tissues of control mice, but demonstrated increased expression after Hdac3 knockout (Fig. 2a). Furthermore, the primary lung fibroblasts were isolated from BPD mice and stably transfected with sh-NC and sh-Hdac3. The western blot analysis demonstrated that the protein level of Hdac3 was notably diminished following transfection of sh-Hdac3 (Fig. 2b). Moreover, the expression of miR-17-92 cluster in sh-Hdac3-treated cells was markedly higher than those of sh-NC-treated cells (Fig. 2c), implying that Hdac3 may inhibit the miR-17-92 cluster in BPD. To further elucidate the enrichment of Hdac3 in the promoter region of the miR-17-92 cluster, ChIP assay was conducted, which showed that Hdac3 was enriched in the miR-17-92 cluster promoter, while silencing of Hdac3 resulted in an opposite trend (Fig. 2d). Furthermore, the dual-luciferase reporter gene assay showed that silencing of Hdac3 resulted in restricted luciferase activity of miR-17-92 promoter (Fig. 2e). Based on the aforementioned results, it was suggested that Hdac3 downregulated the expression of miR-17-92 cluster in BPD.

### EZH1 is a target gene of miR-17 in the miR-17-92 cluster

**Fig. 3:**
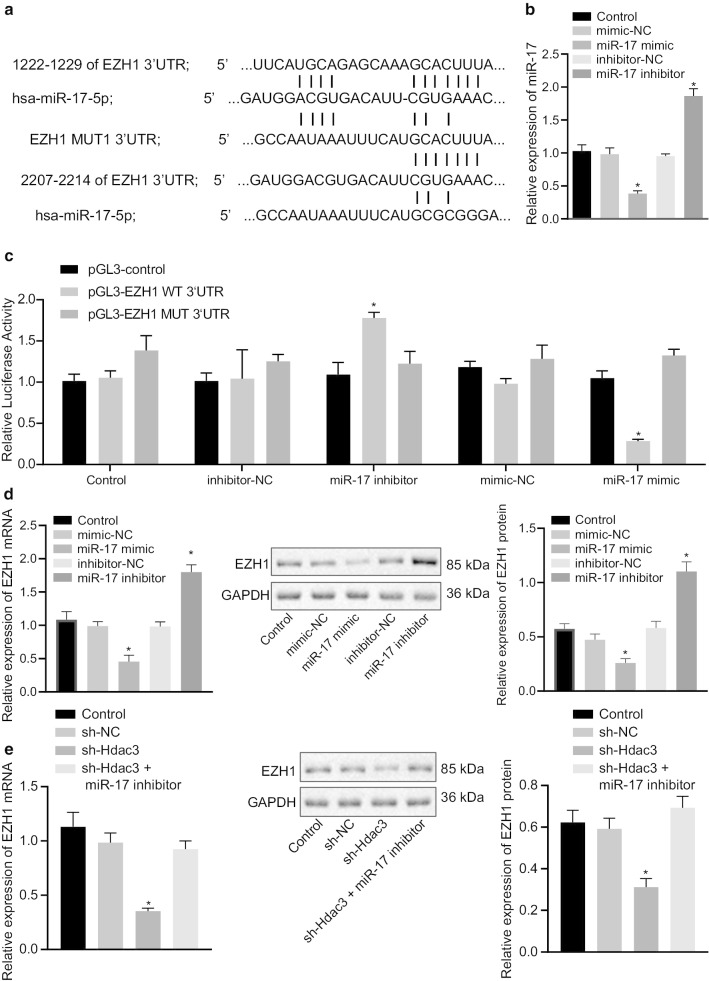
miR-17 in the miR-17-92 cluster targets EZH1 and downregulates its expression in primary lung fibroblasts of BPD mice. **a** The binding site of miR-17 and EZH1 predicted by Targetscan website. **b** The expression of miR-17 in cells transfected with miR-17 mimic and miR-17 inhibitor assayed by RT-qPCR. **c** The binding of miR-17 to EZH1 assayed by dual-luciferase reporter gene assay. **d** The expression of EZH1 in cells transfected with miR-17 mimic and miR-17 inhibitor assayed by RT-qPCR and Western blot analysis. **e** The expression of EZH1 in fibroblasts transfected with sh-Hdac3, or both sh-Hdac3 and miR-17 inhibitor assayed by RT-qPCR and Western blot analysis. *p < 0.05 compared to inhibitor *NC* mimic NC, or sh-NC. The above data were expressed as mean ± standard deviation. Data among multiple groups were analyzed by one-way ANOVA, followed by Tukey’s post-hoc test

This study further explored the underlying mechanism of miR-17 in the miR-17-92 cluster in BPD. The Targetscan website predicted binding sites between miR-17 and EZH1 (Fig. 3a). To further explore the regulatory mechanism of miR-17 and EZH1 in abnormal alveolarization, lung fibroblasts were stably transfected with miR-17 inhibitor or mimic in BPD. RT-qPCR identified that miR-17 inhibitor transfected in lung fibroblasts resulted in diminished miR-17 expression, while miR-17 mimic resulted in increased miR-17 expression (Fig. 3b). The dual-luciferase reporter gene assay revealed that overexpression of miR-17 resulted in restricted luciferase activity of EZH1 3′UTR, while suppression of miR-17 resulted in accelerated luciferase activity of EZH1 3′UTR (Fig. 3c). Results from RT-qPCR and Western blot analysis demonstrated that overexpressed miR-17 resulted in restricted EZH1 expression, whereas downregulated miR-17 resulted in accelerated EZH1 expression (Fig. 3d). Further, we validated that the expression of EZH1 was restricted in the presence of sh-Hdac3 expression, but it could be restored by the co-transfection of sh-Hdac3 and miR-17 inhibitor in fibroblasts (Fig. 3e). The above results showed that miR-17 may be involved in the Hdac3-mediated regulation of EZH1 in BPD.

### EZH1 activates p65 transcription factor to enhance Pgf expression

**Fig. 4:**
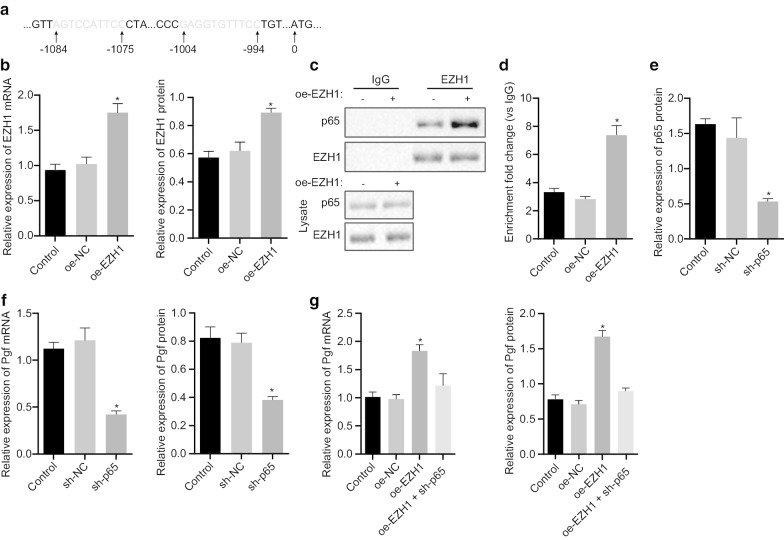
EZH1 elevates the expression of Pgf via stimulating p65 transcription factor expression in primary lung fibroblasts of BPD mice. **a** The binding sites between p65 and Pgf. **b** The expression of EZH1 in cells transfected with oe-EZH1 assayed by RT-qPCR and Western blot analysis. **c** The binding of EZH1 and p65 assayed by immunoprecipitation assay. **d** The enrichment of p65 in the Pgf promoter region in oe-EZH1-treated cells assayed by ChIP assay. **e** The expression of p65 in sh-p65-treated cells assayed by Western blot analysis. **f** The expression of Pgf in sh-p65-treated cells assayed by RT-qPCR and Western blot analysis. **g** The expression of Pgf in fibroblasts transfected with oe-EZH1, or both oe-EZH1 and sh-p65 assayed by RT-qPCR and Western blot analysis. *p < 0.05 compared to oe-NC or sh-NC. The above data were expressed as mean ± standard deviation. The data between the two groups with unpaired design were compared by unpaired *t*-test. Data among multiple groups were analyzed by one-way ANOVA, followed by Tukey’s post-hoc test

After clarifying the interplay of Hdac3/miR-17/EZH1, we shifted to illuminate the EZH1-related downstream mechanistic actions. Initially, the binding sites of p65 and Pgf were obtained (Fig. 4a). RT-qPCR and Western blot analysis revealed that the expression of EZH1 was up-regulated after the transfection with oe-EZH1 in fibroblasts of BPD mice (Fig. 4b). The immunoprecipitation assay validated that the overexpression of EZH1 accelerated the binding of EZH1 to p65 (Fig. 4c). Additionally, results from the ChIP assay revealed that the overexpression of EZH1 accelerated the enrichment of p65 in the Pgf promoter region (Fig. 4d). The transfection of sh-p65 in non-fibroblasts of BPD mice restricted the expression of p65 (Fig. 4e). Meanwhile, silencing of p65 reduced the expression of Pgf (Fig. 4f). Moreover, it has been indicated that EZH1 may be involved in the regulation of Pgf expression. Next, we verified that Pgf expression was appreciably elevated in oe-EZH1-treated cells, and the expression of Pgf was rescued in cells after co-transfection of sh-p65 and oe-EZH1 (Fig. 4g). The above-mentioned data indicated that EZH1 may participate in the regulation of BPD by recruiting p65 to augment Pgf expression.

### Hdac3 upregulates Pgf expression via miR-17-EZH1-p65 axis

**Fig. 5:**
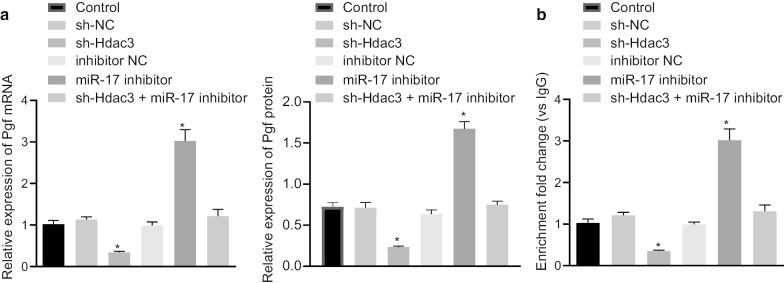
Hdac3 potentiates the transcription and the expression of Pgf via the miR-17-EZH1-p65 axis in primary lung fibroblasts of BPD mice. **a** The expression of Pgf assayed by RT-qPCR and Western blot analysis in cells transfected with sh-Hdac3, miR-17 inhibitor or both. **b** The enrichment of p65 in Pgf promoter region assayed by ChIP assay in cells transfected with sh-Hdac3, miR-17 inhibitor or both. *p < 0.05 compared to sh-NC or inhibitor NC. The above data were expressed as mean ± standard deviation. Data among multiple groups were analyzed by one-way ANOVA, followed by Tukey’s post-hoc test

Next, we sought to validate the regulatory role of Hdac3 on the miR-17-EZH1-p65 axis in BPD. RT-qPCR and western blot analysis suggested that the miR-17 inhibitor resulted in accelerated Pgf expression, whereas sh-Hdac3 resulted in restricted Pgf expression, while Pgf expression was rescued in cells after co-transfection of sh-Hdac3 and miR-17 inhibitor. In addition, Pgf protein expression was not significantly increased in cells co-transfected with sh-Hdac3 and miR-17 mimic compared to control cells (Fig. 5a). Based on our previous results, Hdac3 upregulated the expression of EZH1 through miR-17, therefore it was hypothesized that Hdac3 may augment the transcription of Pgf via miR-17-EZH1-p65 axis. ChIP assay showed that silencing of Hdac3 restricted the enrichment of p65 in the Pgf promoter region while silencing of miR-17 accelerated the enrichment of p65 in the Pgf promoter region. Additionally, the co-transfection of sh-Hdac3 and miR-17 inhibitor restored the enrichment of p65 in the Pgf promoter region (Fig. 5b). These results demonstrated that Hdac3 enhanced the transcription and expression of Pgf via impairment of miR-17-regulated EZH1 inhibition and subsequently augment the recruitment of p65 in the Pgf promoter region.

### Hdac3 upregulates Pgf via miR-17 downregulation in miR-17-92 cluster and promotes BPD development

**Fig. 6:**
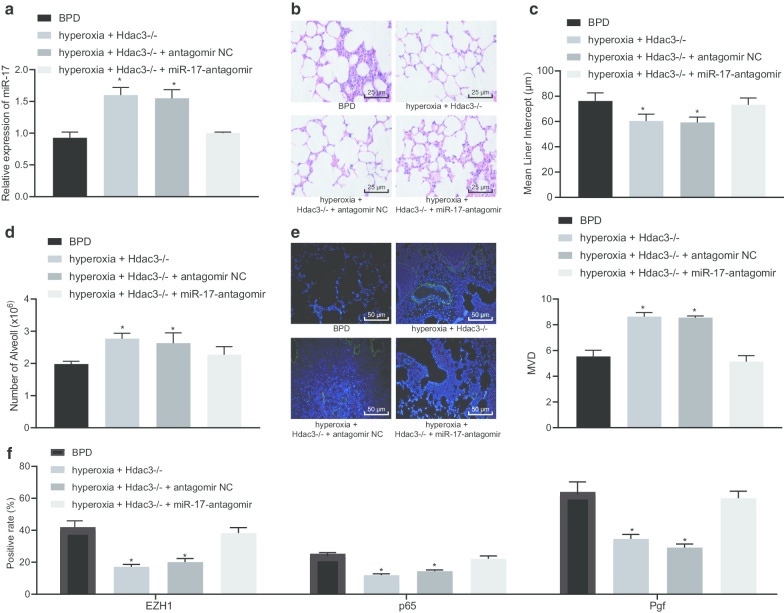
Hdac3 upregulates Pgf expression through miR-17 inhibition to augment the abnormal alveolarization and pulmonary angiogenesis of BPD mice. **a** The expression of miR-17 in lung tissues of mice in different groups assayed by RT-qPCR. **b** The cell morphology in lung tissues of mice of different groups assayed by HE staining (× 400). **c** The MLI of alveolar in the lung tissues of mice in each group. **d** The changes in alveolar number in lung tissues of mice in each group. **e** Immunofluorescence assay of MVD in lung tissues of mice in each group (× 200). **f** The quantitation of expression of EZH1, p65, and Pgf in lung tissues of mice following different treatments assayed by immunohistochemistry. *p < 0.05 compared to untreated BPD mice. # *p* < 0.05 compared to mice subjected to hyperoxia + Hdac3^−/−^ + antagomir NC. The above data were expressed as mean ± standard deviation. Data among multiple groups were analyzed by one-way ANOVA, followed by Tukey’s post-hoc test. *n* = 10 mice for each group

To further demonstrate the regulatory role of Hdac3 on Pgf expression in BPD mice via the miR-17-EZH1-p65 axis in vivo, hyperoxia + Hdac3^−/−^ + miR-17-antagomir mice were constructed through injection of miR-17 antagomir into hyperoxia + Hdac3^−/−^ mice. The RT-qPCR results showed that miR-17 expression in lung tissues of hyperoxia + Hdac3^−/−^ mice was higher than that in lung tissues of hyperoxia-induced BPD mice, but it was diminished in lung tissues of hyperoxia + Hdac3^−/−^ + miR-17-antagomir-treated mice, which was similar to that in lung tissues of the BPD mice (Fig. 6a). Furthermore, HE staining analysis demonstrated that the abnormal cell morphology was improved in hyperoxia + Hdac3^−/−^-treated mice when compared to the hyperoxia-induced BPD mice. The abnormality of cell morphology in hyperoxia + Hdac3^−/−^ + miR-17-antagomir-treated mice was similar to that in the hyperoxia-induced BPD mice (Fig. 6b). In terms of the degree of abnormal alveolarization in mice, the MLI of hyperoxia + Hdac3^−/−^-treated mice was reduced, and that of the hyperoxia + Hdac3^−/−^ + miR-17-antagomir mice was similar to that of hyperoxia-induced BPD mice (Fig. 6c). The number of alveolar in hyperoxia + Hdac3^−/−^-treated mice was notably higher than that in the BPD mice, while the number in hyperoxia + Hdac3^−/−^ + miR-17-antagomir-treated mice was similar to that in the hyperoxia-induced BPD mice (Fig. 6d). The immunofluorescence assay showed that the MVD of hyperoxia + Hdac3^−/−^-treated mice was markedly higher than that of BPD mice, and the MVD of hyperoxia + Hdac3^−/−^ + miR-17-antagomir-treated mice was similar to that of the hyperoxia-induced BPD mice (Fig. 6E). Additionally, it was shown that Hdac3 advanced the abnormal alveolarization and angiogenesis via miR-17 in BPD mice. Furthermore, immunohistochemistry identified that the expression of EZH1, p65, and Pgf in lung tissues of hyperoxia + Hdac3^−/−^-treated mice was lower than that in BPD mice, whereas the expression of EZH1, p65, and Pgf in lung tissues of hyperoxia + Hdac3 ^−/−^ + miR-17-antagomir-treated mice was similar to the hyperoxia-induced BPD mice (Fig. 6f, Additional file 1: Fig. S1). The above-mentioned results demonstrated that Hdac3 upregulated Pgf expression through the miR-17-EZH1-p65 axis to enhance abnormal alveolarization and pulmonary angiogenesis of BPD mice.

## Discussion

BPD is a serious complication afflicting preterm infants which arises from oxygen toxicity and mechanical injury during oxygen supplementation (Jobe 2016). Existing literature has highlighted the participation of HDAC in various pathological and physiological processes including BPD (Cantley et al. 2017). In this study, we established in vivo BPD mouse models and identified that Hdac3 might be an endogenous regulator of BPD, as Hdac3 augmented the abnormal angiogenesis and alveolarization in BPD by enhancing Pgf via the miR-17-EZH1-p65 axis.

Initial results revealed that the expression of Hdac3 was highly expressed while the miR-17-92 cluster was poorly expressed in BPD mice. As one of the members in the class I histone deacetylase family, Hdac3 possesses regulatory function on gene expression through the deacetylation of both histones and non-histone proteins (Chini et al. 2010). Hdac3 has been shown to be upregulated in adenocarcinoma of the lung, which was related to its poor prognosis (Minamiya et al. 2010). In agreement with our findings, the expression of miR-17 in the miR-17–92 cluster has been found to be poorly expressed in infants diagnosed with severe BPD, and also was correlated with the diagnosis of BPD (Robbins et al. 2016). Previous evidence has shown that Hdac3 could participate in the regulation of the remodeling and expansion of distant alveolar vesicles into primitive lung alveologenesis through inhibition of miR-17-92 expression (Wang et al. 2016), suggesting that Hdac3 may exert a regulatory role in the abnormal angiogenesis and alveolarization of BPD through inhibiting the expression of miR-17-92 cluster.

Next, the results obtained from dual-luciferase reporter gene assay indicated that miR-17 could bind to the 3′-UTR of EZH1, demonstrating that EZH1 was a target gene of miR-17 and was negatively regulated by miR-17. EZH1, a component of polycomb repressive complex 2, has been demonstrated to exert a significant role in repressing the transcription of target genes that affect the pathogenesis of various diseases (Rizq et al. 2017). The interaction between miRNA and its target mRNAs and their functional mechanisms in the pathophysiology of BPD have been addressed (Dong et al. 2012). EZH1 has been previously reported to be a target gene of miR-17-5p, and the down-regulation of miR-17-5p could enhance the resistance to erlotinib in non-small cell lung cancer by upregulating EZH1 (Zhang et al. 2017), which was in consistent with our experimental results whereby miR-17 in the miR-17-92 cluster negatively regulated EZH1 expression, which reflects the biological characteristics of BPD.

Subsequently, we found out that EZH1 augmented Pgf expression by recruiting p65 and then participated in the regulation of BPD. As an important molecule in angiogenesis, Pgf/Plgf has a regulatory effect on pathological conditions including tumor formation, ischemia, cardiovascular diseases, suggesting that it may serve as a novel therapeutic target in various diseases (Sorice et al. 2012). Moreover, the evidence provided by Procianoy et al*.* has documented that the levels of Pgf were elevated in preterm neonates suffering from BPD (Procianoy et al., 2016), indicative of a potential association between Pgf and pathophysiological process of BPD. Pgf mRNA expression was found predominantly expressed by the vasculosyncytial membrane of villous trophoblast, and maternal decidual cells showed strong staining for Pgf immunoreactive protein (Khaliq et al., 1996); therefore, Pgf is essential for the angiogenesis of placental chorionic membrane, and also for the embryonic development. Based on previous literature, it was demonstrated that EZH1 could mediate the recruitment of p65 to augment the transcription of target genes (Su et al. 2016). In addition, p65 could directly bind to the Pgf promoter region, leading to a substantial activation of Pgf transcript levels (Cramer et al. 2005). These results were in line with our results and the interaction of EZH1, p65 and Pgf in regulating the abnormal angiogenesis and alveolarization of BPD were verified. Another important finding was that Hdac3 could stimulate Pgf expression via the miR-17-EZH1-p65 axis, thus promoting the progression of BPD.

## Conclusion

**Fig. 7:**
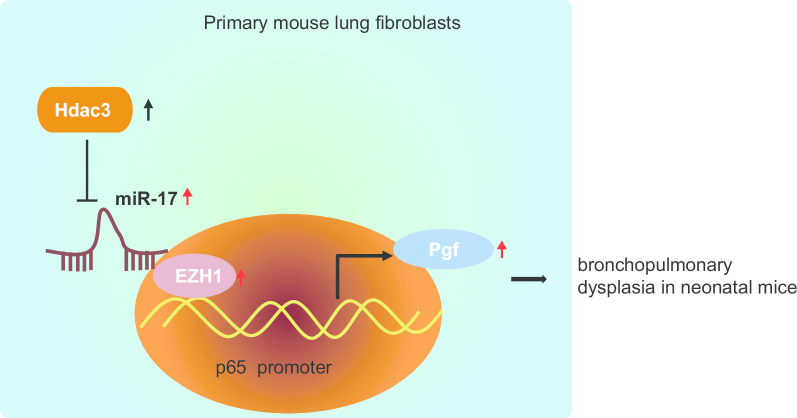
Schematic diagram depicting the role of Hdac3 regulating the miR-17-EZH1-p65-Pgf axis in BPD in neonatal mice. Hdac3 is highly expressed in lung fibroblasts of BPD mice, inhibiting the expression of miR-17 in the miR-17-92 cluster. In addition, Hdac3 attenuates the binding of miR-17 to EZH1, and consequently upregulates the transcription and expression of Pgf, a downstream target gene of transcription factor p65. This thereby promotes angiogenesis and alveolar formation, and aggravates the progression of BPD

In conclusion, the key findings obtained from the current study identified the promotive role of Hdac3 interacting with the miR-17-EZH1-p65-Pgf axis in abnormal pulmonary angiogenesis and alveolarization in newborn mice with BPD (Fig. 7). The upregulation of Hdac3 and inhibition of miR-17 expression were observed in BPD mice. Additionally, Hdac3 could attenuate the binding of miR-17 to EZH1, and consequently enhanced the transcription and expression of the p65 downstream target Pgf, thereby aggravating BPD. These findings suggest that Hdac3 may serve as a promising diagnostic biomarker and therapeutic target for BPD. At present, the effects and mechanisms of Hdac3 interacting with miR-17-EZH1-p65-Pgf in BPD remain scantly identified. Therefore, more in-depth investigation is urgently needed to further discuss the underlying rules that govern their interaction, which might increase the feasibility and safety of its therapy in clinical applications.

### Limitations

The current study only focused on the mouse model of BPD, and it is also recommended that future studies employ specimens from BPD-diagnosed patients for the in-depth analysis of the Hdac3-miR-17-EZH1-p65-Pgf axis in BPD. Moreover, in this axis, whether Hdac3 could stimulate Pgf expression in a direct manner would also require investigation due to the lack of available literature. Evidence for the regulation of Pgf contributing overall to the development of BPD is less compelling and needs further studies. In addition, it is not really clear how increased Pgf expression regulates abnormal angiogenesis associated with BPD if a key characteristic of BPD is delayed (or dysregulated) pulmonary vascular development since there is complex interaction with other mediators of angiogenesis, and also depends on the timing and source of measurement. Meanwhile, we cannot exclude the involvement of other miRs in the miR-17-92 cluster in the abnormal pulmonary angiogenesis and alveolarization in newborn mice with BPD due to the complex microenvironments and the interaction of EZH1 with other miRs.

## Supplementary information

**Additional file 1: Fig. S1.** Representative images of immunohistochemistry (× 400) showing expression of EZH1, p65, and Pgf in lung tissues of mice following different treatments

## Data Availability

The datasets generated and/or analyzed during the current study are available from the corresponding author on reasonable request.

## Abbreviations

BPD: Bronchopulmonary dysplasia
HDACs: Histone deacetylases
miR: MicroRNA
Pgf: Placental growth factor
EZH1: Enhancer of zeste homolog 1
DMEM: Dulbecco's modified Eagle's medium
FBS: Fetal bovine serum
P1: Postnatal day 1
Hdac3^−^^/^^−^: Hdac3 knockout
NC: Negative control
sh: Short hairpin
oe: Overexpression
HE: Hematoxylin–eosin
SP: Streptavidin-peroxidase
MVD: Microvessel density
RT-qPCR: Reverse transcription-quantitative polymerase chain reaction
cDNA: Complementary DNA
GAPDH: Glyceraldehyde-3-phosphate dehydrogenase
TBST: Tris-buffered saline Tween-20
SDS-PAGE: Sodium dodecyl sulfate–polyacrylamide gel electrophoresis
HRP: Horseradish peroxidase
ChIP: Chromatin immunoprecipitation
NaCl: Sodium chloride
DAB: 3,3′-Diaminobenzidine tetrahydrochloride
MVD: Microvessel density
PBS: Phosphate-buffered saline
EDTA: Ethylenediamine tetraacetic acid
PIC: Pseudoisocyanine
IgG: Immunoglobulin G
3′UTR: 3′Untranslated region
ANOVA: Analysis of variance
MLI: Mean linear intercept
